# Prevalence of alcohol consumption among clients of opioid agonist treatment (OAT) centers in Golestan province, Iran

**DOI:** 10.3389/fpsyt.2023.1134683

**Published:** 2023-05-25

**Authors:** Alireza Noroozi, Ahmad Danesh

**Affiliations:** ^1^Iranian National Center for Addiction Studies (INCAS), Tehran University of Medical Sciences (TUMS), Tehran, Iran; ^2^Health Management and Social Development Research Center, School of Medicine, Golestan University of Medical Sciences, Gorgan, Golestan, Iran

**Keywords:** alcohol, opioid agonist treatment (OAT), opioid use disorder (OUD), methadone, buprenorphine

## Abstract

**Introduction:**

Opioid agonist treatments (OATs) with methadone and buprenorphine are known to be effective treatments for people with opioid use disorder (OUD). However, concomitant use of other substances such as alcohol can negatively affect OAT outcomes. This study aimed to determine the prevalence of alcohol use among clients of OAT centers in the Golestan province in the northern part of Iran.

**Materials and methods:**

This is a secondary analysis of a sample of 706 clients who were receiving OATs from certified OAT centers in Golestan province in 2015. They had been on OATs for at least 1 month and were randomly selected for the study. Data were collected via interviews with selected OAT clients. The main indicators studied in the present study were lifetime history of alcohol consumption, alcohol consumption during last month, lifetime history of excessive alcohol use on one occasion, and years of regular alcohol consumption.

**Results:**

The prevalence of lifetime history of alcohol consumption was estimated at 39.2%. Prevalence of alcohol consumption during last month and lifetime history of excessive alcohol use on one occasion was 6.9 and 18.8%, respectively.

**Conclusion:**

Despite a total ban on alcohol consumption in Iran, a sub-sample of participants admitted past-month alcohol use concurrent with their OATs. The estimated past-month prevalence of alcohol use was lower than the reported prevalence in countries where the production, distribution, and consumption of alcohol are legal.

## Introduction

Opioid agonist treatments (OATs) with long-acting opioid medications including methadone and buprenorphine are safe and effective treatments for people with moderate to severe opioid use disorder (OUD) (1). The ultimate advantage of using OATs could be reducing death, particularly from overdoes, and criminal activities (2–4). It is estimated that one-third of those on OATs have concurrent alcohol use disorder (AUD) or harmful drinking (5, 6). This is based on studies conducted in different parts of the world and in different treatment settings (7–9). Although, alcohol use does not appear to change with OAT, concomitant alcohol use with opioid agonist medications is often problematic and can lead to a range of adverse consequences including hepatotoxicity, cirrhosis, intoxication, and overdose (10–14). In addition, unhealthy alcohol consumption has been identified as a risk factor for non-adherence to OAT, problems in social life, criminal activities, and illicit drug use (5, 6, 15–17). Moreover, drinking alcohol may exacerbate mental health problems of those receiving OAT (18, 19).

Alcohol use disorder and its physical and psychological effects should be considered when providing OAT services to people with OUD (20). International studies have shown that screening and brief motivational intervention, could effectively reduce hazardous or harmful drinking and improve treatment outcomes among OAT clients (21). Therefore, professional staff working in OAT centers can play a crucial role in early detection and management of problematic alcohol use in this population (22).

International studies have shown the prevalence of alcohol use up to 30% among people receiving OATs, but this data is mainly from countries where drinking alcohol is legal and culturally acceptable (5). However, the prevalence could be quite different in Muslim-majority countries such as Iran, Afghanistan, Pakistan, and Saudi Arabia, where there is complete ban on the production, sale, and consumption of alcohol (23). Results from a recent study in Iran show the overall pooled prevalence of lifetime alcohol consumption among the general population in 31% (24).

To answer this question, we analyzed data from previous research on the simultaneous use of amphetamines and opioids among clients of OAT centers to estimate the prevalence of alcohol consumption among these clients (15). Therefore, this is a secondary analysis on a random sample of 706 clients attending in OAT centers in Golestan province.

## Materials and methods

The initial study was a cross-sectional study on a random sample of 706 clients referred to outpatient OAT centers in Golestan province, in the northern part of Iran. The detailed methods of the study were reported elsewhere (15).

### Procedure

In brief, the participants were selected by two-stage cluster sampling with two strata based on the location of the centers (Gorgan and the other cities of Golestan province). In the first stage, 25 centers were randomly selected from all 150 centers in Golestan province. Then, in the second stage, 30 clients of the selected centers were recruited through convenience sampling. Inclusion criteria were at least 18 years old at the time of the interview, receiving OAT with methadone, buprenorphine, or opium tincture for at least 1 month prior to the interview, and providing informed consent to participate in the study.

A questionnaire designed by researchers was used to collect the data (15). Face-to-face interviews were conducted to collect information on drug and alcohol use history (i.e., lifetime history of alcohol consumption, alcohol consumption during the last month, lifetime history of excessive alcohol use on one occasion, and years of regular alcohol consumption). Excessive alcohol consumption was defined as consumption of 6 standard drinks of alcohol or more containing 10 grams or 12.7 ml of pure alcohol on one occasion. Years of regular alcohol consumption was defined as number of years in which the participants were drinking three or more times in a week. Age, gender, employment status, monthly income, level of education, type of OAT medication (methadone, buprenorphine, or opium tincture), duration of OAT, and history of imprisonment were among the variables collected in the initial study. These variables were used as possible factors related to alcohol consumption. To confirm the participants’ response to the recent drug use, urine samples were collected from the participants to identify their status regarding recent consumption of morphine, tramadol, amphetamine, methamphetamine, cannabis (THC), and benzodiazepines. Information about alcohol consumption was based on self-report.

### Data analysis

The analyses of this study were performed by survey data analysis commands using STATA software (version 10). Descriptive statistics were calculated for demographic characteristics, type of OAT, and imprisonment history of the study participants. Results for the prevalence of alcohol consumption were presented by point estimate and 95% confidence interval. To examine possible correlated factors, a multiple logistic regression model was used and the adjusted odds ratio and 95% confidence interval of the estimates were calculated. Sex, age, education, type of OAT, treatment duration, and history of imprisonment were included in the model using the forward method. All data analysis were two-tailed, and the results with a *p*-value of < 0.05 was considered to be statistically significant.

Participation in the study was completely voluntary and the participants provided written informed consent to participate in this study. The study protocol and questionnaire were approved by the ethics committee of the Golestan University of Medical Sciences GOUMS (code: IR.GOUMS.REC.1394.112).

## Results

A sample of 706 OAT clients was include in the study. Table 1 shows demographic characteristics, socioeconomic status, type and duration of OAT, and history of imprisonment of the study participants. The majority of participants were male (93.6%) and most of them were on methadone maintenance treatment (89.2%). The prevalence of lifetime alcohol consumption was 39.2% (95% CI, 29.5–48.9). Alcohol consumption during last month and lifetime history of excessive alcohol use on one occasion were 6.9% (95% CI, 3.8–10.0) and 18.8% (95% CI, 12.2–25.4), respectively. Average number of days of alcohol consumption in people who had consumed alcohol in the past month was 1 day (0.94: 95% CI, 0.44–1.4). Only 8 of the 45 female participants (18%) reported lifetime drinking. None of them had consumed alcohol in the 30 days prior to the interview. The mean age of alcohol consumption initiation was 20.8 years (95% CI, 19.7–21.9) and the average years of regular alcohol consumption was 5 years among those who admitted lifetime alcohol use (95% CI, 4–6).

**TABLE 1:** Demographic characteristics, type of OAT (opioid agonist treatment), and imprisonment history of the study participants (706 people; 661 men and 45 women).

Variable	Mean (SE)/Percent
**Sex**	
Male	93.6
Female	6.4
Age (year)	39.9 (0.44)
**Education**	
Up to diploma	61.7
Diploma and above	38.3
**Employment**	
Full-time	31.7
Part-time	44
Unemployed	24.3
**Income (per month)**	
Less than 50,000 Toman	24.8
50,000 toman and above	75.2
**Type of OAT**	
Methadone	89.2
Buprenorphine	9.1
Opium tincture	1.7
Treatment duration (month)	26.7 (2.1)
**History of imprisonment**	
Yes	22.6
No	77.4

Prevalence of lifetime history of alcohol consumption for participants receiving methadone, buprenorphine, and opium tincture treatments were 41.9% (95% CI, 33.1–50.8), 17.2% (95% CI, 3.3–31.1), and 40% (95% CI, 18.5–61.4), respectively. It seems that there is no difference between methadone and buprenorphine groups in terms of lifetime history of alcohol consumption, alcohol consumption during last month, and lifetime history of excessive alcohol use in one occasion (Table 2).

**TABLE 2:** Prevalence of alcohol consumption history (lifetime history of alcohol consumption, alcohol consumption during the last month, lifetime history of excessive alcohol use on one occasion) based on the type of OAT (i.e., methadone, buprenorphine, opium tincture).

Type of OAT	Lifetime history of alcohol consumption	Alcohol consumption during the last month	Lifetime history of excessive alcohol use on one occasion
Methadone	41.9 (33.1–50.8)	7.3 (3.8–10.8)	20.2 (13.9–26.6)
Buprenorphine	17.2 (3.3–31.1)	2.8 (0.0–6.5)	7.0 (0.0–16.2)
Opium tincture	40.0 (18.5–61.4)	14.1 (0.0–31.4)	20.0 (0.0–48.8)

Data are point estimate and 95% confidence interval of lifetime history of alcohol consumption, alcohol consumption during last month, and lifetime history of excessive alcohol use on one occasion from 706 OAT clients disintegrated by the type of OAT (i.e., methadone, buprenorphine, opium tincture). OAT, opioid agonist treatment.

Table 3 shows the prevalence of alcohol consumption by demographic characteristics, socioeconomic status, type and duration of treatment, and imprisonment history. Those who reported alcohol consumption (lifetime, past month, and lifetime excessive alcohol consumption) were younger and had a higher level of education. Duration of regular alcohol consumption in people with higher levels of education (high school diploma and above) was significantly longer than people with lower level of education. It was 6.3 years (95% CI, 4.4–8.1) for people with a higher level of education and 3.8 years (95% CI, 3.2–4.5) for a lower level of education. People with lifetime history of alcohol consumption were more likely to report lifetime incarceration. People who were on methadone maintenance treatment or had a history of incarceration were more likely to report lifetime history of alcohol consumption. Those who reported lifetime history of excessive alcohol consumption on one occasion were more likely to have a history of incarceration. In the multivariate logistic model only history of imprisonment was significantly associated with lifetime history of alcohol consumption (OR = 3.2 with 95% confidence interval; 2.1–4.9), alcohol consumption during last month (OR = 2.3 with 95% confidence interval; 1.4–3.7), and lifetime history of excessive alcohol use on one occasion (OR = 3.9 with 95% confidence interval; 2.3–6.7).

**TABLE 3:** Prevalence of alcohol consumption (lifetime, during the last month) and lifetime history of excessive alcohol consumption by demographic characteristics, socioeconomic status, type and duration of OAT, and prison history.

Variable	Lifetime history of alcohol consumption		*P*-value	Alcohol consumption during the last month		*P*-value	Lifetime history of excessive alcohol use on one occasion		*P*-value
	Yes	No		Yes	No		Yes	No	
**Sex**									
Male	278 (42.6)	375 (57.4)	0.237	44 (6.7)	612 (93.3)	0.318	136 (20.7)	520 (97.3)	0.001
Female	8 (18.2)	36 (81.8)		0 (0.0)	45 (100.0)		1 (2.2)	44 (97.8)	
**Age (year)**	37.5 (35.8–39.2)	41.9 (40.2–43.6)	0.000	35.0 (32.7–37.3)	40.5 (39.0–42.1)	0.001	36.3 (33.9–38.7)	41.0 (39.5–42.6)	0.000
**Education**									
Up to high school diploma	154 (35.6)	279 (64.4)	0.025	12 (2.8)	423 (97.2)	0.000	70 (16.1)	365 (83.9)	0.034
High school diploma and above	133 (49.6)	135 (50.4)		32 (11.9)	238 (88.1)		67 (24.8)	203 (75.2)	
**Employment**									
Full-time	100 (45.1)	122 (54.9)	0.289	19 (8.5)	204 (91.1)	0.055	40 (17.9)	183 (82.1)	0.362
Unemployed, Part-time job or other sources of income	186 (39.1)	290 (60.9)		25 (5.2)	454 (94.8)		97 (20.3)	382 (79.7)	
**Income (per month)**									
Less than 50,000 Toman	70 (42.4)	95 (57.6)	0.861	6 (3.6)	159 (96.4)	0.012	33 (20.0)	132 (80.0)	0.978
50,000 toman and above	198 (39.8)	299 (60.2)		37 (7.4)	463 (92.6)		96 (19.2)	404 (80.8)	
**Type of OAT**									
Methadone	268 (42.9)	356 (57.1)	0.003	41 (6.5)	587 (93.5)	0.186	129 (20.5)	499 (79.5)	0.111
Buprenorphine	14 (21.9)	50 (78.1)		2 (3.1)	62 (96.9)		5 (7.8)	59 (92.2)	
Opium tincture	5 (41.7)	7 (58.3)		1 (8.3)	11 (91.7)		3 (25.0)	9 (75.0)	
Treatment duration (month)	28.8 (15.9–41.7)	25.2 (20–30.4)	0.325	19.7 (12.1–27.3)	27.0 (19.6–34.3)	0.457	33.1 (15.6–52.3)	24.8 (19.2–30.4)	0.036
**History of imprisonment**									
Yes	90 (57.0)	68 (43.0)	0.000	11 (7.0)	147 (93.0)	0.450	54 (34.2)	104 (65.8)	0.001
No	193 (35.9)	345 (64.1)		33 (6.1)	509 (93.9)		81 (14.9)	461 (85.1)	

Data are number (present) from 706 OAT clients who responded to three questions; lifetime history of alcohol consumption, alcohol consumption during last month, lifetime history of excessive alcohol use on one occasion by t-test and Chi-squared test.

Table 4 presents prevalence of lifetime history of alcohol consumption, alcohol consumption during last month, and lifetime history of excessive alcohol use on one occasion based on participants’ rapid urine test results for morphine, amphetamines, cannabis, benzodiazepines, and tramadol. The prevalence of alcohol consumption was the same among those with positive rapid urine tests of morphine, cannabis, or tramadol. Participants who tested positive for amphetamines included 7.6% of the sample (with 95% confidence interval; 1.4–13) and reported higher lifetime history of alcohol consumption and lifetime history of excessive alcohol use on one occasion as compared to participants who tested negative for amphetamines. Although the difference between these two groups regarding lifetime history of excessive alcohol use on one occasion was statistically significant, the 95% confidence intervals had some overlap (Table 3). In addition, this group had a significantly longer duration of regular alcohol consumption than those who tested negative for amphetamines: 7.3 years (with 95% confidence interval; 9.1–5.6) compared to 4.7 years (with 95% confidence interval; 5.6–3.8). Participants whose urine test was positive for benzodiazepines expressed more alcohol consumption during last month than those whose urine test was negative for benzodiazepines (*p* = 0.038).

**TABLE 4:** Prevalence of alcohol consumption (lifetime, last month) and lifetime excessive alcohol consumption based on participants’ urine test results for morphine, amphetamines, THC, benzodiazepines, and tramadol.

Substance test result	Lifetime history of alcohol consumption	*P*-value	Alcohol consumption during last month	*P*-value	Lifetime history of excessive alcohol use on one occasion	*P*-value
**Morphine**						
Yes	42.9 (34.2–51.5)	0.106	6.7 (0.0–13.7)	0.658	19.9 (13.3–26.5)	0.638
No	35.7 (23.0–48.5)		5.5 (2.4–8.6)		18.0 (8.9–27.1)	
**Amphetamines**						
Yes	61.4 (44.4–78.5)	0.005	5.1 (1.0–9.3)	0.482	34.8 (21.2–48.4)	0.001
No	37.4 (28.1–46.6)		7.0 (3.8–10.3)		17.5 (11.6–23.3)	
**Cannabis**						
Yes	48.5 (32.0–65.0)	0.119	3.4 (0.0–8.0)	0.418	23.7 (4.5–42.8)	0.609
No	37.5 (26.8–48.2)		6.4 (1.6–11.2)		18.3 (10.3–26.4)	
**Benzodiazepines**						
Yes	36.7 (27.2–46.2)	0.437	9.2 (1.6–16.8)	0.038	19.2 (10.9–27.4)	0.947
No	40.6 (26.9–54.3)		3.4 (1.4–5.4)		18.9 (11.3–26.6)	
**Tramadol**						
Yes	46.9 (29.1–64.6)	0.290	13.2 (0.0–29.7)	0.044	28.3 (13.8–42.7)	0.139
No	37.5 (26.3–48.7)		4.9 (2.1–7.7)		9.8 (9.8–25.2)	

Data are point estimate and 95% confidence interval of lifetime history of alcohol consumption, alcohol consumption during last month, and lifetime history of excessive alcohol use on one occasion from 706 OAT clients disintegrated by the type of urine toxicology test results.

## Discussion

This study aimed to determine the prevalence of alcohol consumption among clients of OAT centers using data from previous study. Estimates were based on data collected from a random sample of 706 clients referred to outpatient centers for OAT in Golestan province.

The prevalence of self-reported past month alcohol consumption in our study (6.9% with 95% confidence interval: 3.8–10.0) was lower than OAT clients in countries where alcohol is legally produced and consumed (5, 22, 25, 26).

Among those who admitted to the past month alcohol drinking, the average number of alcohol-drinking day was 1 day in a range from 0.44 to 1.4 days which show a low frequency of alcohol use during the last month. It is even less than the frequency of alcohol consumption in the EU general population (27).

One possible explanation for the low prevalence of alcohol use in our sample might be due to the self-reported nature of our data. Self-report as the method of data collection could result in underreporting due to social desirability bias (28). This possibility can be further strengthened, especially by considering the illegality of alcohol production and consumption in Iran, as well as heavy penalties for producers and consumers of alcohol (29). Although it should be noted that, according to Iran’s national protocols for OAT concurrent alcohol drinking is not associated with any negative effect on continuation of OAT services, rather treatment providers are recommended to provide more intensive services for such clients if their level of drinking would be at problematic levels (30). It should be noted that the reported indicators in our study do not provide needed data to estimate the prevalence of problematic alcohol use or alcohol use disorder. Further studies to measure the prevalence of alcohol use disorders using standard screening questionnaires or interviews are warranted.

Like studies on the general population, we found that OAT clients with a history of alcohol consumption were younger and had higher levels of education (31–33). The next point is the relationship between alcohol consumption and prison history, which may indicate an association between alcohol use and criminal activities in this sample (33). Further studies to explore the nature of this association are warranted. Moreover, alcohol consumption during the last month was higher among people who had a positive rapid urine test for benzodiazepines. It might be due to the concomitant use of these substances or adding benzodiazepines to alcoholic beverages by sellers in the illicit alcohol market as a cheap strategy to increase their intoxicating effects (34).

We found low alcohol consumption among clients of OAT centers in Golestan province, which is far lower than the figures provided by countries without legal restrictions on alcohol consumption. This finding does not necessarily indicate a low level of alcohol consumption in the general population but may suggest that people who use alcohol are probably a different class of people who use other substances in Iran. According to the latest finding in Iran, the overall pooled prevalence of alcohol consumption during the last 12-month, among the general population is 12% (24).

The relationship between alcohol consumption and some individual and social factors such as use of amphetamines and a history of imprisonment should lead treatment professionals to further examine and screen these people for alcohol consumption at treatment admission. It is also necessary for physicians to consider concomitant use of benzodiazepines and alcohol at the beginning of therapy because this can have a great impact on determining the dose required for the induction phase of OAT. According to the findings of this study and the previous study by the same group (15), it is recommended that these clients must first be screened for the possibility of using several substances before starting to treatment. It is also recommended to have such evaluation during the treatment process.

## Data availability statement

The raw data supporting the conclusions of this article will be made available by the authors, without undue reservation.

## Ethics statement

This study involving humans was approved by the Ethic Committee of Golestan University of Medical Sciences GOUMS (code: IR.GOUMS.REC.1394.112). The participants provided their written informed consent to participate in this study.

## Author contributions

Both authors made contribution to the concept and design of the article, analysis, and interpretation of data, drafted and revised the manuscript critically, reviewed the results, and approved the final version of the manuscript.
